# Incidence of herpes zoster and associated events including stroke—a population-based cohort study

**DOI:** 10.1186/s12879-015-1170-y

**Published:** 2015-10-31

**Authors:** Karin Sundström, Caroline E. Weibull, Karin Söderberg-Löfdal, Tomas Bergström, Pär Sparén, Lisen Arnheim-Dahlström

**Affiliations:** Department of Laboratory Medicine, Karolinska Institutet, S-141 86 Stockholm, Sweden; Department of Medical Epidemiology and Biostatistics, Karolinska Institutet, S-141 86 Stockholm, Sweden; Division of Clinical Pharmacology, Dept. of Laboratory Medicine, Karolinska Institutet, S-141 86 Stockholm, Sweden; Department of Infectious Diseases, Institute of Biomedicine, University of Gothenburg, S-405 30 Gothenburg, Sweden

**Keywords:** Herpes zoster, Incidence, Cohort, Stroke, Sepsis

## Abstract

**Background:**

More detailed understanding of herpes zoster (HZ) is called for in the context of an increasing observed frequency of disease, and ongoing discussions regarding potential consequences of the disease. Thus, population-based data on incidence and complications of HZ are needed.

**Methods:**

We conducted a register-based cohort study in Västra Götaland County (population 1.5 million) in Sweden. We collected data on all patients diagnosed with HZ during the years 2008 to 2010 from population-based registers. Incidence rates (IR) of HZ and related complications were calculated and stratified by age and sex.

**Results:**

There were 13 269 new HZ cases during the study period. Overall, the IR of herpes zoster in both genders was 3.25 (95 % CI: 3.16–3.34) per 1000 person years in 2010. The incidence was consistently higher in women than in men and in older than in young. A history of immunosuppression was more common than in the general population. The incidence was highest in individuals over 80 years of age (IR 9.2 per 1000 person years, 95 % CI: 8.8–9.6) during 2008–2010. The most common complications to HZ were ocular engagement and postherpetic neuralgia; risks for stroke and sepsis were significantly elevated during the one year following diagnosis, especially in the youngest age group of patients 0–39 years.

**Conclusions:**

Herpes zoster is more common in women, the elderly and immunosuppressed individuals. We verify a population-based association between herpes zoster and risk for stroke and sepsis, which may merit concern.

**Electronic supplementary material:**

The online version of this article (doi:10.1186/s12879-015-1170-y) contains supplementary material, which is available to authorized users.

## Background

Herpes zoster, also called shingles, is a spontaneous reactivation of a latent infection with varicella zoster virus (VZV). Primary infection with VZV causes chickenpox, a common and typically benign exanthematous childhood rash, after which VZV becomes latent in nerve ganglia. The lifetime risk of re-activation is about 20–30 % [1, 2]. Re-activation of VZV usually manifests as herpes zoster with pain, rash, and vesicles in the area of skin supplied by the affected nerve (dermatome). The risk increases with age, as host cell-mediated immunity declines, and herpes zoster is similarly also more common in immunosuppressed patients. The most common complication related to herpes zoster, especially among the elderly, is the pain syndrome post-herpetic neuralgia (PHN), typically defined as pain persisting more than 90 days [3], which can cause severe distress. Other complications of herpes zoster include bacterial skin infection, encephalitis, meningitis, keratitis and herpes zoster ophtalmicus, the latter of which has been associated with a risk of developing stroke [4, 5]. A possible biological explanation for this association is the viral ability to replicate in cerebral arteries where the infection is spread along the nerve fibers to the blood vessels and thrombotic responses are induced [6]. Regarding bacterial involvement after VZV infection, this has been observed as a risk factor for invasive disease and long-term complications [7].

VZV vaccination is not part of the child vaccination program in Sweden and VZV is therefore a common infection in children. Over 90 % of the Swedish population will have had chickenpox before 12 years of age, which is more common than in southern Europe [8] and corresponds to about a birth cohort every year (~100 000/year in Sweden). With a viral reactivation of 20–30 %, an extensive number of individuals are likely to be affected each year in the population, although not everyone will seek healthcare. Thus, estimation of real-life incidence can be challenging, although such information is increasingly needed as the introduction of VZV-vaccination has taken place or is being contemplated in a number of countries [9]. Therefore, in this study we investigated herpes zoster incidence, complications, associated events, and the economic burden incurred; using population-based health care registers in Sweden.

## METHODS

### Study population

We conducted a cohort study using population-based health care registers in Västra Götaland County (VGC) in Sweden. VGC is the second largest county in Sweden and covers a stable population of approximately 1.5 million inhabitants. Our study cohort consisted of all incident cases of herpes zoster in this region during years 2008 to 2010. The VGC Primary Health Care Register (PHCR) includes records for all visits to general practitioners in the county since 2000. The Swedish Patient Register (SPR) records all in- and outpatient care visits to hospitals and is complete in this aspect since 2001. These two registers were used to find all registered incident cases of herpes zoster (ICD-10: B02) occurring in the VGC population during the study years. The SPR was also used to identify complications related to herpes zoster, as this register holds more detailed information on such compared to the PHCR, and to identify potential associated outcomes after herpes zoster as well as underlying immunosuppressive conditions. Using the Swedish personal identification number, which is unique to each individual, we linked our cohort of herpes zoster patients to other registers held at the Swedish National Board of Health and Welfare. The National Cancer Registry was used to identify patients with a cancer diagnosis prior to herpes zoster, and the Swedish Prescribed Drug Register (PDR) was used to collect dispensation information on drugs associated with treatment for herpes zoster, and immunosuppressive drugs (see below).

### Definitions of cases and complications

An incident case of herpes zoster was defined as receipt of a diagnosis of herpes zoster (ICD-10: B02) during 2008–2010 in an individual with no diagnosis of herpes zoster during the preceding year. PHN was defined as having a combination of either ICD-10 code B02.2 and G53.3, or B02 and G63.0 (either one of these as main/secondary diagnosis) on the same date of diagnosis. Other herpes zoster-related complications were defined as receipt of a combination of ICD-10 code B02 diagnosis together with the ICD-10 code for the respective complication/-s (Additional file 1: Table S1). A potentially associated adverse event of either cardiovascular disease (CVD excluding stroke), stroke, sepsis or Bell’s palsy was defined as receipt of a diagnosis of such, according to the respective ICD-10 code (Additional file 1: Table S1) within one year following herpes zoster diagnosis. Immunosuppressed patients were defined as incident herpes zoster cases that had received either a prescription of an immunosuppressive drug (Additional file 1: Table S2) within 90–365 days prior to diagnosis of herpes zoster, or a diagnosis of one of the following immunosuppressive conditions (IC): HIV/AIDS, organ transplantation, or any type of cancer (ICD10 C00-C99), within one or five years prior to diagnosis (Additional file 1: Table S1).

### Case validation

To investigate the validity of a recorded diagnosis of herpes zoster in the VGC PHCR and the SPR, we performed a nested validation study. A random sample of 112 cases was selected for the validation set. For these patients, requests for copies of medical charts were sent to the diagnosing clinic. All records were de-identified at the clinic before review.

A clinician (KS) then reviewed all charts according to a set of pre-defined criteria, to determine whether each case of herpes zoster could be considered as a verified case or not (Additional file 1: Table S3).

### Statistical analyses

Incidence rates (IRs) and 95 % confidence intervals (CI) of herpes zoster per 1,000 person-years were calculated as the ratio of the number of incident cases to the total amount of person-time at risk in the county. The rates were both stratified by age at diagnosis (0–49/50–54/…75–79/80+) as well as age-standardized to the Swedish population in 2010, and further stratified by calendar year of diagnosis and sex. IRs for herpes zoster-related complications were calculated in a similar manner as for herpes zoster, but expressed per 100,000 person-years.

The PHCR only uses the ICD-10 code B02, regardless of whether the individual presents with any herpes zoster-related complications. As the ICD-10 for PHN (G53) was not available in this register, to study pain complications we used a combination of appropriate ICD-10 codes with complementary information from the PDR on analgesics against neuropathic pain. We calculated the frequency and proportion of herpes zoster patients with a PHN diagnosis, and/or a prescription for analgesics against neuropathic pain, at the time of herpes zoster diagnosis, or within specified following time windows. We considered both inclusive windows up to 90 days (≤30, ≤60, ≤90 days) as well as exclusive windows up to 1 year after diagnosis (0–30, 30–60, 60–90, 90–180, and 180–365 days).

To quantify the association between herpes zoster and potentially related adverse events such as stroke, patients were followed for 1 year after diagnosis of herpes zoster, within which all main diagnoses of the selected diseases were recorded, as well as all person-time at risk. Patients continued to be at risk for the full year, irrespective of whether an adverse event occurred. Poisson regression models were used to compare patients with herpes zoster to the general population in VGC by estimating incidence rate ratios (IRRs) with 95 % CIs. All models were adjusted for sex and age, and in a second step the effect was allowed to vary by sex and age by the inclusion of interaction terms in the model.

### Cost calculations

Overall costs for herpes zoster health care visits during 2010 for Västra Götaland County were estimated. Calculations included all health care visits with ICD-code B02 as the main diagnosis and, using costing data from the PDR, also all antiviral, ocular and specific analgesic drugs purchased during 2010 within 90 days after receiving a main diagnosis of herpes zoster. In order to enable this 90-day follow up for all patients, 3992 individuals diagnosed between January-September of 2010 were included in these calculations. These individuals were not required to be incident cases only; prevalent cases were also included. Direct costs related to herpes zoster were calculated from unit costs using regional county council prices: 940 SEK per primary health care visit, 2082 SEK per outpatient visit and 6313 SEK per day for inpatient care. 1 Euro was calculated as equivalent to 9.43 SEK.

The study was approved by the Ethical Review Board of Stockholm, which determined that informed consent from the participants was not required.

## Results

### Case validation

A total of 73 % of the randomly sampled herpes zoster cases could be directly verified through chart review (81/112) as having ≥3 out of 5 pre-specified symptoms (Additional file 1: Table S3). An additional fifteen cases (13 %) were given a status of probable herpes zoster diagnosis in the validation. These cases were mainly difficult to verify due to only short notes such as “typical herpes zoster”. Thus, the verification proportion was 86 %. Thirteen cases (12 %) were found to have an unclear/unlikely diagnosis of herpes zoster, frequently due to the diagnosing clinician having recorded uncertainty about the correct diagnosis, or an atypical symptom. Only 3 cases (3 %) were found to have a clearly incorrect diagnosis.

### Incidence of herpes zoster

**Fig. 1:**
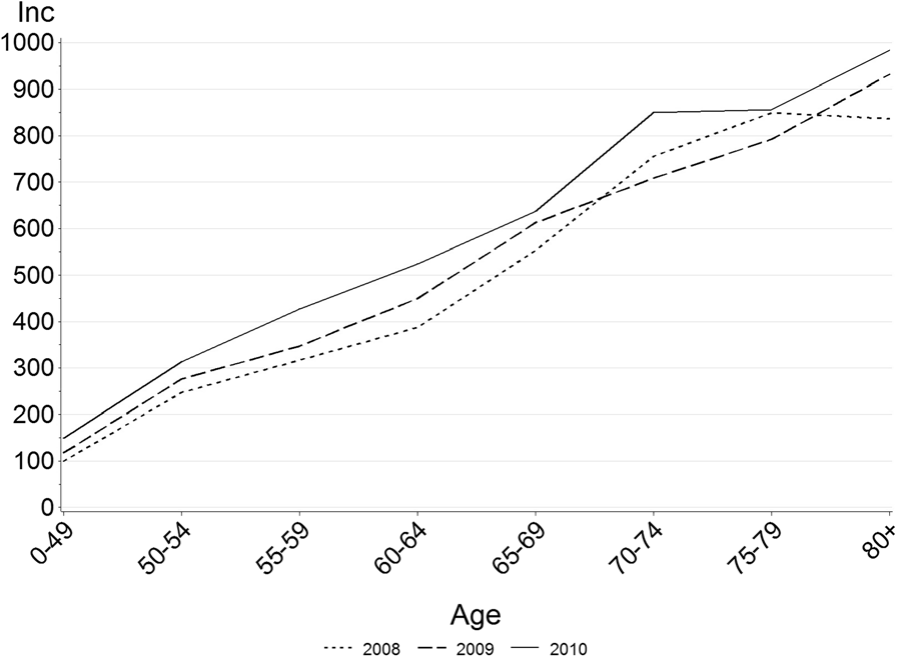
Incidence of herpes zoster per 100 000 person-years in Västra Götaland County during year 2008–2010, by age and calendar year

In total, 13 296 incident cases of herpes zoster were recorded in Västra Götaland County during the years 2008–2010 (Table 1). The number of new cases increased per year (Additional file 1: Table S4), against a background population that remained stable in size (Additional file 1: Table S5). The overall incidence of herpes zoster was 2.6 (95 % CI: 2.5–2.7) in 2008, 2.8 (95 % CI: 2.7–2.9) in 2009 and 3.3 (95 % CI: 3.2–3.3) cases per 1000 person years in 2010 (Table 2). The incidence was consistently higher in women than in men. Gradually increasing with age, the incidence was highest in those over 80 years (Fig. 1 and Table 2).

**Table 1:** Age- and sex distribution of herpes zoster cases in Västra Götaland County during year 2008–2010

		Men		Women		Both sexes	
	Age	N	%	N	%	N	%
2008–2010	0–49	1,656	31.1	1,979	24.8	3,635	27.3
	50–54	277	5.2	545	6.8	822	6.2
	55–59	391	7.3	662	8.3	1,053	7.9
	60–64	560	10.5	819	10.3	1,379	10.4
	65–69	631	11.8	818	10.3	1,449	10.9
	70–74	571	10.7	838	10.5	1,409	10.6
	75–79	503	9.4	769	9.7	1,272	9.6
	80+	739	13.9	1,538	19.3	2,277	17.1
		5,328	100	7,968	100	13,296	100

**Table 2:** Incidence rates (IR) per 1,000 person-years of herpes zoster by sex, age and year

		Men		Women		Both sexes	
	Age	N	IR (95 % CI)	N	IR (95 % CI)	N	IR (95 % CI)
2008–2010	0–49	1,656	1.1 (1.0–1.1)	1,979	1.4 (1.3–1.4)	3,635	1.2 (1.2–1.3)
	50–54	277	1.9 (1.7–2.1)	545	3.7 (3.4–4.1)	822	2.8 (2.6–3.0)
	55–59	391	2.7 (2.4–3.0)	662	4.6 (4.2–4.9)	1,053	3.6 (3.4–3.9)
	60–64	560	3.7 (3.4–4.0)	819	5.4 (5.1–5.8)	1,379	4.5 (4.3–4.8)
	65–69	631	5.3 (4.9–5.7)	818	6.8 (6.3–7.2)	1,449	6.0 (5.7–6.3)
	70–74	571	6.6 (6.0–7.1)	838	8.8 (8.2–9.4)	1,409	7.7 (7.3–8.1)
	75–79	503	7.4 (6.8–8.1)	769	9.1 (8.4–9.7)	1,272	8.3 (7.9–8.8)
	80+	739	8.0 (7.4–8.6)	1,538	9.9 (9.4–10.4)	2,277	9.2 (8.8–9.6)
2008	Total	1,595	2.1^a^ (2.0–2.2)	2,319	3.0^a^ (2.9–3.2)	3,914	2.6^a^ (2.5–2.7)
	0–49	449	0.9 (0.8–1.0)	530	1.1 (1.0–1.2)	979	1.0 (0.9–1.1)
	50–54	81	1.6 (1.3–2.0)	161	3.3 (2.8–3.9)	242	2.5 (2.2–2.8)
	55–59	131	2.7 (2.2–3.2)	180	3.7 (3.2–4.3)	311	3.2 (2.8–3.5)
	60–64	168	3.3 (2.8–3.8)	223	4.4 (3.9–5.1)	391	3.9 (3.5–4.3)
	65–69	178	4.8 (4.1–5.5)	240	6.3 (5.5–7.1)	418	5.5 (5.0–6.1)
	70–74	183	6.6 (5.6–7.6)	263	8.5 (7.5–9.5)	446	7.6 (6.9–8.3)
	75–79	185	8.1 (7.0–9.4)	251	8.8 (7.7–10.0)	436	8.5 (7.7–9.3)
	80+	220	7.2 (6.3–8.2)	471	9.1 (8.3–9.9)	691	8.4 (7.8–9.0)
2009	Total	1,719	2.2^a^ (2.1–2.4)	2,606	3.4^a^ (3.2–3.5)	4,325	2.8^a^ (2.7–2.9)
	0–49	538	1.1 (1.0–1.2)	628	1.3 (1.2–1.4)	1,166	1.2 (1.1–1.2)
	50–54	89	1.8 (1.4–2.2)	182	3.7 (3.2–4.3)	271	2.8 (2.4–3.1)
	55–59	109	2.3 (1.9–2.7)	226	4.7 (4.1–5.3)	335	3.5 (3.1–3.9)
	60–64	190	3.7 (3.2–4.3)	268	5.3 (4.7–6.0)	458	4.5 (4.1–4.9)
	65–69	209	5.3 (4.6–6.0)	281	7.0 (6.2–7.9)	490	6.1 (5.6–6.7)
	70–74	179	6.2 (5.3–7.1)	252	7.9 (7.0–9.0)	431	7.1 (6.4–7.8)
	75–79	158	7.0 (5.9–8.2)	245	8.7 (7.6–9.8)	403	7.9 (7.2–8.7)
	80+	247	8.0 (7.0–9.1)	524	10.1 (9.3–11.0)	771	9.3 (8.7–10.0)
2010	Total	2,014	2.6^a^ (2.5–2.7)	3,043	3.9^a^ (3.8–4.0)	5,057	3.3^a^ (3.2–3.3)
	0–49	669	1.3 (1.2–1.4)	821	1.7 (1.6–1.8)	1,490	1.5 (1.4–1.6)
	50–54	107	2.1 (1.8–2.6)	202	4.1 (3.6–4.8)	309	3.1 (2.8–3.5)
	55–59	151	3.2 (2.7–3.7)	256	5.4 (4.7–6.1)	407	4.3 (3.9–4.7)
	60–64	202	4.0 (3.4–4.6)	328	6.5 (5.8–7.3)	530	5.2 (4.8–5.7)
	65–69	244	5.7 (5.0–6.5)	297	7.0 (6.2–7.8)	541	6.4 (5.8–6.9)
	70–74	209	7.0 (6.1–8.0)	323	9.9 (8.9–11.0)	532	8.5 (7.8–9.3)
	75–79	160	7.1 (6.0–8.3)	273	9.8 (8.6–11.0)	433	8.6 (7.8–9.4)
	80+	272	8.8 (7.7–9.9)	543	10.5 (9.6–11.4)	815	9.8 (9.2–10.5)

^a^Age-standardized to the Swedish population 2010. Rates represent herpes zoster in Västra Götaland County, defined as all recorded cases of B02 ICD-10 codes as main or secondary diagnosis

### Pain related to herpes zoster

**Fig. 2:**
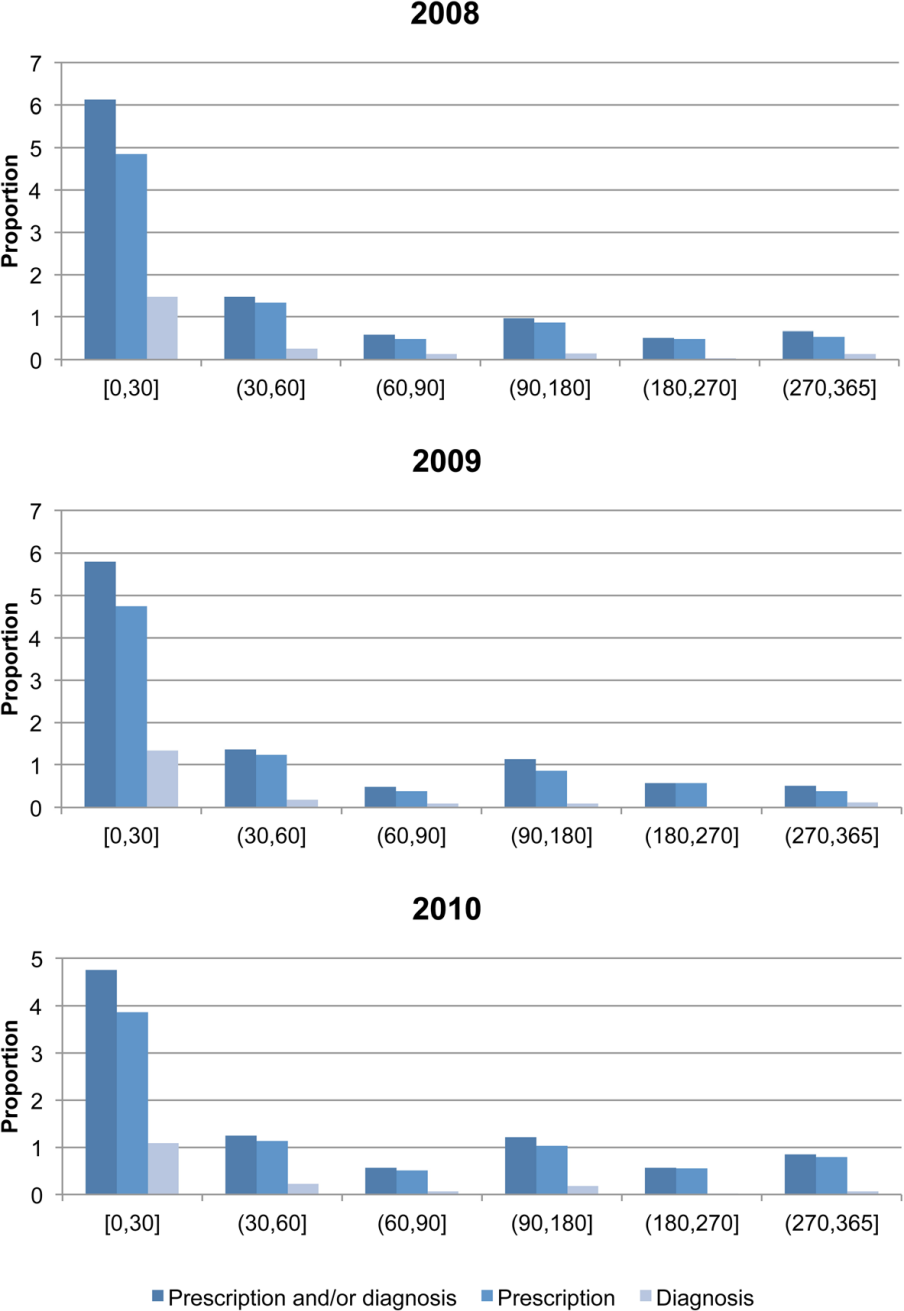
Proportion of cases with prescription of analgesics against neuropathic pain, or diagnosis of PHN

The proportion of all cases who received analgesics against neuropathic pain within 90 days from diagnosis was 5.5–6.7 % during 2008–2010. No substantial gender differences in prescription patterns were discernible (Table 3). In each time window, an additional 0.5–1 percentage unit of all remaining cases were also issued such analgesics (Fig. 2). In terms of explicit diagnosis of PHN, in applicable cases this was most commonly done in conjunction with the herpes zoster diagnosis—i.e. within 0–30 days—and was then a relatively rare event in the following year (Fig. 2). Ultimately, 8.8 % of all men and 9.5 % of all women with herpes zoster had received analgesics and/or a diagnosis of PHN, at 1 year after diagnosis (2010 data, not shown). This proportion varied from 4 % in the age group 0–49 years, to 14.5 % in the oldest age group above 80 years (2010 data, not shown).

**Table 3:** Frequency and proportion of herpes zoster cases with prescription of analgesics and/or PHN diagnosis

	2008			2009			2010		
	Total number of HZ cases 3,914			Total number of HZ cases 4,325			Total number of HZ cases 5,057		
	n (%)			n (%)			n (%)		
	30 days	60 days	90 days	30 days	60 days	90 days	30 days	60 days	90 days
Prescription^a^									
All	190 (4.9)	242 (6.2)	261 (6.7)	205 (4.7)	258 (6.0)	275 (6.4)	195 (3.9)	525 (5.0)	278 (5.5)
Sex									
Male	65 (4.1)	89 (5.6)	96 (6.0)	85 (4.9)	107 (6.2)	114 (6.6)	83 (4.1)	103 (5.1)	113 (5.6)
Female	125 (5.4)	153 (6.6)	165 (7.1)	120 (4.6)	151 (5.8)	161 (6.2)	112 (3.7)	149 (4.9)	165 (5.4)
Age									
0–49	19 (1.9)	21 (2.2)	22 (2.3)	16 (1.4)	21 (1.8)	26 (2.2)	18 (1.2)	27 (1.8)	29 (2.0)
50–54	5 (2.1)	8 (3.3)	10 (4.1)	11 (4.1)	13 (4.8)	13 (4.8)	7 (2.3)	10 (3.2)	13 (4.2)
55–59	14 (4.5)	14 (4.5)	14 (4.5)	16 (4.8)	24 (7.2)	24 (7.2)	14 (3.4)	17 (4.2)	18 (4.4)
60–64	12 (3.1)	13 (3.3)	15 (3.8)	19 (4.2)	22 (4.8)	22 (4.8)	21 (4.0)	23 (4.3)	24 (4.5)
65–69	22 (5.3)	28 (6.7)	30 (7.2)	25 (5.1)	33 (6.7)	34 (6.9)	24 (4.4)	33 (6.1)	36 (6.7)
70–74	23 (5.2)	28 (6.3)	31 (7.0)	22 (5.1)	27 (6.3)	30 (7.0)	34 (6.4)	43 (8.1)	47 (8.8)
75–79	34 (7.8)	45 (10.3)	47 (10.8)	32 (7.9)	38 (9.4)	39 (9.7)	29 (6.7)	36 (8.3)	37 (8.6)
80+	61 (8.8)	85 (12.3)	92 (13.3)	64 (8.3)	80 (10.4)	87 (11.3)	48 (5.9)	63 (7.7)	74 (9.1)
PHN diagnosis^b^									
All	58 (1.5)	68 (1.7)	73 (1.9)	58 (1.3)	66 (1.5)	70 (1.6)	55 (1.1)	66 (1.3)	69 (1.4)
Sex									
Male	21 (1.3)	24 (1.5)	26 (1.6)	32 (1.9)	36 (2.1)	38 (2.2)	21 (1.0)	25 (1.2)	27 (1.3)
Female	37 (1.6)	44 (1.9)	47 (2.0)	26 (1.0)	30 (1.2)	32 (1.2)	34 (1.1)	41 (1.4)	42 (1.4)
Age									
0–49	6 (0.6)	7 (0.7)	7 (0.7)	13 (1.1)	14 (1.2)	14 (1.2)	12 (0.8)	13 (0.9)	13 (0.9)
50–54	1 (0.4)	2 (0.8)	2 (0.8)	3 (1.1)	4 (1.5)	4 (1.5)	3 (1.0)	3 (1.0)	3 (1.0)
55–59	4 (1.3)	5 (1.6)	5 (1.6)	6 (1.8)	6 (1.8)	7 (2.1)	2 (0.5)	2 (0.5)	2 (0.5)
60–64	2 (0.5)	3 (0.8)	3 (0.8)	2 (0.4)	3 (0.7)	3 (0.7)	6 (1.1)	7 (1.3)	8 (1.5)
65–69	5 (1.2)	6 (1.4)	6 (1.4)	3 (0.6)	4 (0.8)	4 (0.8)	5 (0.9)	6 (1.1)	6 (1.1)
70–74	3 (0.7)	5 (1.1)	6 (1.4)	5 (1.2)	5 (1.2)	6 (1.4)	2 (0.4)	4 (0.8)	5 (0.9)
75–79	8 (1.8)	9 (2.1)	12 (2.8)	4 (1.0)	7 (1.7)	7 (1.7)	7 (1.6)	9 (2.1)	10 (2.3)
80+	29 (4.2)	31 (4.5)	32 (4.6)	22 (2.9)	23 (3.0)	25 (3.2)	18 (2.2)	22 (2.7)	22 (2.7)
Prescription^a^ and/or diagnosis^b^									
All	240 (6.1)	294 (7.5)	313 (8.0)	251 (5.8)	305 (7.1)	322 (7.5)	240 (4.8)	298 (5.9)	325 (6.4)
Sex									
Male	81 (5.1)	105 (6.6)	113 (7.1)	112 (6.5)	134 (7.8)	140 (8.1)	100 (5.0)	122 (6.1)	134 (6.7)
Female	159 (6.9)	189 (8.2)	200 (8.6)	139 (5.3)	171 (6.6)	182 (7.0)	140 (4.6)	176 (5.8)	191 (6.3)
Age									
0–49	25 (2.6)	28 (2.9)	29 (3.0)	27 (2.3)	33 (2.8)	38 (3.3)	29 (2.0)	37 (2.5)	39 (2.6)
50–54	6 (2.5)	10 (4.1)	12 (5.0)	13 (4.8)	16 (5.9)	16 (5.9)	10 (3.2)	13 (4.2)	16 (5.2)
55–59	18 (5.8)	19 (6.1)	19 (6.1)	22 (6.6)	28 (8.4)	28 (8.4)	16 (3.9)	19 (4.7)	20 (4.9)
60–64	14 (3.6)	15 (3.8)	17 (4.4)	21 (4.6)	24 (5.2)	24 (5.2)	26 (4.9)	28 (5.3)	30 (5.7)
65–69	27 (6.5)	33 (7.9)	35 (8.4)	26 (5.3)	35 (7.1)	36 (7.4)	25 (4.6)	34 (6.3)	37 (6.8)
70–74	26 (5.8)	31 (7.0)	34 (7.6)	25 (5.8)	30 (7.0)	33 (7.7)	36 (6.8)	45 (8.5)	49 (9.2)
75–79	41 (9.4)	53 (12.2)	55 (12.6)	35 (8.7)	42 (10.4)	43 (10.7)	33 (7.6)	40 (9.2)	42 (9.7)
80+	83 (12.0)	105 (15.2)	112 (16.2)	82 (10.6)	97 (12.6)	104 (13.5)	65 (8.0)	82 (10.1)	92 (11.3)

N (%) = Frequency (proportion) among total number of herpes zoster patients who had a prescription/diagnosis. ^a^According to ATC codes in Additional file 1: Table S2 ^b^According to diagnosis codes in Additional file 1: Table S1

### Herpes zoster-related complications

The most commonly registered complications were ocular engagement and PHN, representing a total of 4 % and 2 % of all cases, respectively, over the years 2008–2010. Severe complications such as disseminated herpes zoster (0.32 %), zoster-related encephalitis (0.29 %) and zoster-related meningitis (0.14 %) were rare (Table 4). The incidence of complications in 2010 was 11.5/100 000 person-years for ocular engagement, 0.6/100 000 person-years for zoster-related encephalitis and meningitis, and 1.2/100 000 person-years for disseminated disease (data not shown). The incidence of ocular engagement appeared higher in women than in men but the rarity of severe complications precluded meaningful interpretation of differences between sexes, or age groups (data not shown).

**Table 4:** Frequency and proportion of herpes zoster cases with a related complication

	Men	Women	Both sexes
	N (%)	N (%)	N (%)
Herpes zoster (HZ)	5,328 (100)	7,968 (100)	13,296 (100)
HZ + PHN	121 (2.27)	157 (1.97)	278 (2.09)
HZ + ocular	218 (4.09)	325 (4.08)	543 (4.08)
HZ + encephalitis	13 (0.24)	26 (0.33)	39 (0.29)
HZ + meningitis	11 (0.21)	8 (0.10)	19 (0.14)
Disseminated HZ	19 (0.36)	24 (0.30)	43 (0.32)
Other HZ complications	69 (1.30)	89 (1.12)	158 (1.19)
HZ without complications	1,127 (21.2)	1,613 (20.2)	2,740 (20.6)

### Potentially associated events after herpes zoster

A total of 212 cases of CVD, stroke and sepsis occurred within one year of herpes zoster diagnosis, in the cohort of herpes zoster cases. The most frequent of these (*n* = 111) was stroke. Compared to the general population, the age- and sex-adjusted IRRs for CVD, stroke and sepsis were all significantly elevated (Table 5). When allowing the effect to vary between sexes, men had a significantly higher relative incidence of Bell’s palsy (*p* < 0.01). There were no such differences in CVD, stroke or sepsis. However, the IRRs for stroke and sepsis differed by age group (*p* < 0.01), with 10-fold increased IRRs in younger ages, whereas the IRRs in older ages were close to unity (Table 5).

**Table 5:** Incidence rate ratios (IRR) for associated outcomes within a year following herpes zoster diagnosis

	CVD (excl. stroke)		Stroke		Sepsis		Bell’s palsy	
	n	IRR^a^ (95 % CI)	n	IRR^a^ (95 % CI)	n	IRR^a^ (95 % CI)	n	IRR^a^ (95 % CI)
All cases	64	1.82 (1.42–2.33)	111	1.34 (1.12–1.62)	37	1.47 (1.06–2.03)	71	10.6 (8.33–13.4)
Sex								
*Men*	26	1.62 (1.10–2.38)	49	1.30 (0.98–1.72)	15	1.23 (0.74–2.05)	43	15.7 (11.6–21.3)
*Women*	38	1.99 (1.45–2.74)	62	1.38 (1.08–1.77)	22	1.68 (1.11–2.56)	28	6.99 (4.80–10.2)
*p-value* ^b^		0.41		0.75		0.36		<0.01
Age (years)								
*0–39*	0	.	4	10.3 (3.87–27.6)	3	9.10 (2.93–28.3)	15	23.4 (14.0–38.9)
*40–49*	1	1.15 (0.16–8.16)	0	.	3	9.42 (3.02–29.3)	1	2.62 (0.37–18.6)
*50–59*	12	3.51 (1.99–6.19)	2	0.44 (0.11–1.76)	0	.	6	7.23 (3.23–16.2)
*60–69*	21	2.68 (1.75–4.12)	13	1.12 (0.65–1.92)	3	0.79 (0.25–2.44)	17	9.09 (5.61–14.7)
*70–79*	16	1.37 (0.84–2.25)	35	1.47 (1.05–2.04)	6	0.79 (0.35–1.76)	13	7.69 (4.42–13.4)
*80+*	14	1.29 (0.76–2.18)	57	1.38 (1.07–1.80)	22	1.82 (1.19–2.77)	19	14.4 (9.03–23.0)
*p-value* ^c^		0.06		<0.01		<0.01		0.01

^a^Adjusted for age and sex

^b^From Wald test of interaction between HZ and sex

^c^From Wald test of interaction between HZ and age group

### Immunosuppression and herpes zoster

During the study period, 819 of all cases (6.2 %) had a prescription for an immunosuppressive drug within 90 days before diagnosis of herpes zoster. This proportion increased with age: from 5.2 % in those 0–49 years to 12.6 % in those above 80 years (data not shown). Depending on age group, 8.9 %–18.4 % had had a prescription of immunosuppressive medication within the preceding year, compared to only 5.2–15.1 % of the general population in the same five-year age groups from 0–49 to 80+. Furthermore, within the preceding year, 3.2 % of all male cases and 2.2 % of all female cases of herpes zoster had received a diagnosis of an immunosuppressive condition. When considering the previous five years, these proportions increased to 8.2 % and 6.8 %, respectively. The majority of these derived from a previous diagnosis of any cancer (data not shown).

### Costs and health care utilization related to herpes zoster

The costs in year 2010 for VGC inpatient hospital stays, outpatient visits and primary health care visits, incurred by herpes zoster as a main diagnosis, amounted to approximately 1.05 million Euro. More than half of the cost (0.55 million Euro) was incurred in primary health care, closely followed by inpatient care (0.42 million Euro) whereas outpatient care had the lowest related cost (0.08 million Euro). The total cost to VGC and its patients for the above described herpes zoster-related drug prescriptions was 0.28 million Euro. Patients with complicated herpes zoster had relatively higher use of in- and outpatient services than those with uncomplicated disease. They also had higher use of drugs; most apparent for analgesics against neuropathic pain, which was prescribed to only 1 in 5 of uncomplicated cases, but in 1 of 2 complicated cases of herpes zoster, on average (data not shown).

## Discussion

We investigated the burden of herpes zoster disease in a population-based register study of one of the largest counties in Sweden between the years 2008 and 2010. The overall incidence for herpes zoster was 2.6–3.3 cases per 1000 person-years, ranging from 1.2–9.2 cases per 1000 person-years, depending on age group. The healthcare costs in 2010 directly related to the diagnosis amounted to at least 1.33 million Euro for the county of Västra Götaland, where 17 % of the nation’s inhabitants live. In accordance with other studies, we observed an increase in herpes zoster incidence over the years of study [10–12], which in some countries has been attributed to the introduction of vaccination against VZV. As the incidence of chickenpox decreases in vaccinating countries, the elderly will not be exposed to a natural booster by their grandchildren, which may increase later herpes zoster incidence in older birth cohorts. This theory could not, however, explain our findings, since VZV vaccination is not part of the child vaccination program in Sweden. Our observed increase could therefore at least in part be explained by other factors, such as improved surveillance and reporting of cases to health authorities. An ageing population and an increasing number of immunosuppressed patients as a result of solid organ or bone marrow transplantation, may also serve to explain this increasing trend, which should be further investigated.

Herpes zoster incidence was generally higher in women than in men, as observed previously [10, 13] and in line with another Swedish study, although the use in that study of prescription proxies, and hospitalization data only, entails that comparison of incidence rates is challenging [14]. Whether this gender difference is due to biological mechanisms and/or detection bias is not known.

Although strongly related to increasing age, we further observed that 30 % of our cases were found in patients below 50 years of age, emphasizing the important distinction that herpes zoster is not only a geriatric affliction [10, 13, 15, 16].

The most frequent complications to herpes zoster were pain, Bell’s palsy and ocular problems. Herpes zoster-related meningitis and encephalitis were very rare, with an incidence of less than 1/100 000 person-years. In a study investigating viral DNA/RNA in cerebrospinal fluid from patients with suspected infection and neurological syndromes in Västra Götaland County, the estimated incidence of VZV central nervous system infections was 1.8/100 000 person-years [17], as defined by positive PCR-detection of VZV DNA. Our findings as recorded in the Swedish Patient Register may therefore represent a slight underestimation, likely due to meningitis and encephalitis sometimes being coded as “other meningitis and encephalitis”, without reference to the correct B02 ICD code.

When studying the relation between immunosuppressive conditions (IC) and herpes zoster, we found that 5–13 % of our cases had a prescription of an immunosuppressive drug within 90 days before herpes zoster diagnosis, and 6.8–8.2 % had a previous diagnosis of an IC condition, both of which findings confirm results from a UK study where 8 % of hospitalized herpes zoster cases had underlying IC [18]. The latter observation also mirrors the findings by others on a substantially increased risk of herpes zoster in those with cancer and other immunosuppressive diagnoses [16, 19]. We furthermore found that the preceding use of immunosuppressive drugs was more common in our herpes zoster patients than in the general population, even in the ages below 50 years.

It has been proposed that the broader use of corticoids in children is associated with more common affliction of shingles in the same age group [20]. If this applies also to adults, our findings may lend support to this argument, although it is possible that this association is related instead to a higher relative use of healthcare visits/resources.

The proportion of pain-related complications within one year after diagnosis was up to 15 % in the oldest population. The incidence of long-term post-herpetic neuralgia (PHN) caused by herpes zoster varies between studies depending on study population and definitions used. Pain up to 30 days after diagnosis may reflect only acute pain, and not true PHN [21]. We thus present data per time window since diagnosis, to enable a distinction between acute and long-term pain. We partially defined pain/PHN through prescription proxies, and it is not certain whether these analgesics against neuropathic pain were prescribed specifically for pain related to herpes zoster; or actually for some other neuropathic condition. Nevertheless, we focused on reasonable time spans for prescription, and analgesics in line with Swedish guidelines for herpes zoster [22]. Indeed, our observations are in accordance with previous findings, where the condition develops in 8–14 % of patients [23–25].

Herpes zoster may also be related to more severe subsequent outcomes such as stroke [26], where one potential mechanism could be direct infection of cerebral arteries [27]. We observed an increased risk of both stroke and CVD within a year after herpes zoster, which may be in line with previous reports of VZV-related neurological sequelae [5, 17]. We also found that the youngest population (0–39 years) had a 4-fold higher risk of developing stroke within a year after herpes zoster diagnosis—which may support previous findings of VZV as a risk factor for stroke in children [28–30].

In one study, post-varicella cerebral infarctions in children age 0–6 years were uniformly located to the middle cerebral artery territory [29] and it may be of relevance to investigate in clinical studies whether stroke after herpes zoster might show a similar regional pattern.

Furthermore, bacteremia has been associated with chickenpox/primary VZV infection in children [31, 32]. We observed an overall increased risk for subsequent sepsis in herpes zoster patients and the largest risk increase (9-fold) was again found in the youngest age group, which may indicate that herpes zoster is a risk factor in young individuals, similar to chickenpox. These analyses do not adjust for potential confounders in terms of co-morbidities and therefore should be interpreted with caution. Nevertheless, even if there is no causal relation, it is conceivable that occurrence of herpes zoster could act as a risk marker for subsequent severe disease in vulnerable populations.

Since this is a register-based study, there are some instances where registration may have been imperfect, and our conclusions affected, as described above. However, we conclude after our chart validation that our use of the diagnosis code B02 is highly valid. If at least half of the unclear cases were actually cases of sub-optimally documented herpes zoster, the verification proportion would be around 86 %, a figure reflected in other studies [33]. Our proportion of false positives therefore appears low, and although we did not assess the proportion of false negatives, our observed incidence is in line with the published literature, which should entail that our approach is valid for the proportion of herpes zoster cases which have merited/triggered health care attention.

Regarding costs, we did not consider costs for complications that were not connected to the ICD-code B02; prescriptions >90 days after herpes zoster diagnosis, or additional treatments for IC patients with herpes zoster. Also, our definition of IC was somewhat limited compared to other studies. Other studies who have considered these and other additional factors observe a higher cost related to herpes zoster treatment [34, 35]. Our estimates of costs should therefore be regarded as conservative.

## Conclusion

In summary, herpes zoster is a common disease afflicting the population, particularly women, the elderly, and the immunosuppressed. Consistent signs of potentially severe associated events or complications, now verified in a population-based setting, should prompt further mechanistic studies and investigation into high risk-groups.

## Additional file

Additional file 1 Tables S1-S5(DOCX 47 kb)

## Abbreviations

AIDS: Acquired immunodeficiency syndrome
CI: Confidence interval
CVD: Cerebrovascular disease
HIV: Human immunodeficiency virus
HZ: Herpes zoster
IC: immunosuppressive condition
ICD: International classification of diseases
IR: Incidence rate
PDR: prescribed drug register
PHCR: Primary healthcare register
PHN: Post-herpetic neuralgia
SEK: Swedish krona (Swedish currency)
SPR: Swedish patient register
UK: United Kingdom
VGC: Västra Götaland county
VZV: Varicella zoster virus

## References

[1] Bowsher D (1999). The lifetime occurrence of Herpes zoster and prevalence of post-herpetic neuralgia: A retrospective survey in an elderly population. Eur J Pain.

[2] Brisson M, Edmunds WJ, Law B, Gay NJ, Walld R, Brownell M (2001). Epidemiology of varicella zoster virus infection in Canada and the United Kingdom. Epidemiol Infect.

[3] Johnson RW, Rice AS (2014). Clinical practice. Postherpetic neuralgia. N Engl J Med.

[4] Lin HC, Chien CW, Ho JD (2010). Herpes zoster ophthalmicus and the risk of stroke: a population-based follow-up study. Neurology.

[5] Kang JH, Ho JD, Chen YH, Lin HC (2009). Increased risk of stroke after a herpes zoster attack: a population-based follow-up study. Stroke.

[6] Gilden D, Nagel MA, Mahalingam R, Mueller NH, Brazeau EA, Pugazhenthi S (2009). Clinical and molecular aspects of varicella zoster virus infection. Future Neurol.

[7] Ziebold C, von Kries R, Lang R, Weigl J, Schmitt HJ (2001). Severe complications of varicella in previously healthy children in Germany: a 1-year survey. Pediatrics.

[8] Parment PA, Svahn A, Ruden U, Brakenhielm G, Storsaeter J, Akesson L (2003). Immunogenicity and reactogenicity of a single dose of live attenuated varicella vaccine and a booster dose of measles-mumps-rubella vaccine given concomitantly at 12 years of age. Scand J Infect Dis.

[9] WHO (2014). Varicella and herpes zoster vaccines: WHO position paper. Weekly Epidemiological Record (WER).

[10] Esteban-Vasallo MD, Gil-Prieto R, Dominguez-Berjon MF, Astray-Mochales J, Gil De Miguel A (2014). Temporal trends in incidence rates of herpes zoster among patients treated in primary care centers in Madrid (Spain), 2005–2012. J Infect.

[11] Wu PY, Wu HD, Chou TC, Sung FC (2013). Varicella vaccination alters the chronological trends of herpes zoster and varicella. PloS one.

[12] Yawn BP, Gilden D (2013). The global epidemiology of herpes zoster. Neurology.

[13] Yawn BP, Saddier P, Wollan PC, St Sauver JL, Kurland MJ, Sy LS (2007). A population-based study of the incidence and complication rates of herpes zoster before zoster vaccine introduction. Mayo Clin Proc.

[14] Studahl M, Petzold M, Cassel T (2013). Disease burden of herpes zoster in Sweden--predominance in the elderly and in women - a register based study. BMC Infect Dis.

[15] Weitzman D, Shavit O, Stein M, Cohen R, Chodick G, Shalev V (2013). A population based study of the epidemiology of Herpes Zoster and its complications. J Infect.

[16] Forbes HJ, Bhaskaran K, Thomas SL, Smeeth L, Clayton T, Langan SM (2014). Quantification of risk factors for herpes zoster: population based case–control study. Bmj.

[17] Persson A, Bergstrom T, Lindh M, Namvar L, Studahl M (2009). Varicella-zoster virus CNS disease--viral load, clinical manifestations and sequels. J Clin Virol.

[18] Brisson M, Edmunds WJ (2003). Epidemiology of Varicella-Zoster Virus in England and Wales. J Med Virol.

[19] Habel LA, Ray GT, Silverberg MJ, Horberg MA, Yawn BP, Castillo AL (2013). The epidemiology of herpes zoster in patients with newly diagnosed cancer. Cancer Epidemiol Biomarkers Prev.

[20] Allwinn RB, S Doerr HW (2006). Epidemiology of Herpes Zoster: What has Changed?. Monographs Virol.

[21] Yawn BP (2011). Post-shingles neuralgia by any definition is painful, but is it PHN?. Mayo Clinic proceedings.

[22] Läkemedelsverket. Treatment Recommendation: Pharmacotherapy for herpes simplex-, varicella- and herpes zoster infections. Vol. 4, 2005:34–47.

[23] Edmunds WJ, Brisson M, Rose JD (2001). The epidemiology of herpes zoster and potential cost-effectiveness of vaccination in England and Wales. Vaccine.

[24] Hope-Simpson RE (1975). Postherpetic neuralgia. J R Coll Gen Pract.

[25] Schmader KE (2002). Epidemiology and impact on quality of life of postherpetic neuralgia and painful diabetic neuropathy. Clin J Pain.

[26] Nagel MA, Mahalingam R, Cohrs RJ, Gilden D (2010). Virus vasculopathy and stroke: an under-recognized cause and treatment target. Infect. Disord. Drug Targets.

[27] Nagel M, Ortiz GA (2010). Does herpes zoster ophthalmicus increase the risk of stroke?. Neurology.

[28] Askalan R, Laughlin S, Mayank S, Chan A, MacGregor D, Andrew M (2001). Chickenpox and stroke in childhood: a study of frequency and causation. Stroke.

[29] Miravet E, Danchaivijitr N, Basu H, Saunders DE, Ganesan V (2007). Clinical and radiological features of childhood cerebral infarction following varicella zoster virus infection. Dev. Med. Child Neurol..

[30] Ganesan V, Prengler M, McShane MA, Wade AM, Kirkham FJ (2003). Investigation of risk factors in children with arterial ischemic stroke. Ann. Neurol..

[31] Cameron JC, Allan G, Johnston F, Finn A, Heath PT, Booy R (2007). Severe complications of chickenpox in hospitalised children in the UK and Ireland. Arch. Dis. Child..

[32] Pollard AJ, Isaacs A, Hermione Lyall EG, Curtis N, Lee K, Walters S (1996). Potentially lethal bacterial infection associated with varicella zoster virus. Bmj.

[33] Yawn BP, Wollan P, St SJ (2011). Comparing shingles incidence and complication rates from medical record review and administrative database estimates: how close are they?. Am. J. Epidemiol..

[34] Yawn BP, Itzler RF, Wollan PC, Pellissier JM, Sy LS, Saddier P (2009). Health care utilization and cost burden of herpes zoster in a community population. Mayo Clin. Proc..

[35] Gialloreti LE, Merito M, Pezzotti P, Naldi L, Gatti A, Beillat M (2010). Epidemiology and economic burden of herpes zoster and post-herpetic neuralgia in Italy: a retrospective, population-based study. BMC Infect Dis.

