# Supplementary material for: Incidence of herpes zoster and associated events including stroke—a population-based cohort study
Source: BMC Infect Dis. 2015 Oct 31;15:488. doi: 10.1186/s12879-015-1170-y (PMC4628253; doi:10.1186/s12879-015-1170-y)
Supplement: Additional file 1 — Tables S1-S5(DOCX 47 kb) [file 12879_2015_1170_MOESM1_ESM.docx]

**SUPPLEMENTARY TABLE 1**

| ICD-10 code | Diagnosis |  |
| --- | --- | --- |
| Västra Götaland PHCR | | |
| B02 | Herpes zoster |  |
| National Patient Register | | |
| B02.X | Herpes zoster  (includes shingles and zona) |  |
| B02.2  G53  B02 + G63.0 | Zoster with other nervous system involvement:  Post-herpetic neuralgia  Post-herpetic polyneuropathy |  |
| B02.3  B02 + H03.1  B02 + H13.1  B02 + H22.0  B02 + H19.2  B02 + H19.0 | Zoster ocular disease  Zoster:   - Blepharitis+ - Conjunctivitis+ - Iridocyclitis+ - Iritis+ - Keratitis+ - Keratoconjunctivitis+ - Scleritis+ |  |
| B02.0  B02 + G05.1 | Zoster encephalitis |  |
| B02.1  B02 + G02.0 | Zoster meningitis |  |
| B02.7 | Disseminated zoster |  |
| B02.8 | Zoster with other complications |  |
| B02.9 | Zoster without complications  Zoster NOS |  |
| Associated outcomes | | |
| A40  A41 | Sepsis  *Streptococcus*  *Staphylococcus* |  |
| G51.0 | Bell’spalsy |  |
| I60, I65-I67, I69 | Cerebrovascular disease excl. stroke |  |
| I61-I64 (excluding I62) | Stroke |  |
| G46 | Vascular syndrome of brain in cerebrovascular diseases (zero cases in cohort) |  |
| Immunosuppressive diagnoses | | |
| B20-B24 | HIV/AIDS |  |
| Z94 | Organ transplants |  |
| C00-C99 | Any cancer from the cancer register |  |

Suppl. Table 1. ICD codes used for definition of exposure and outcomes.

**SUPPLEMENTARY TABLE 2**

| ATC code | Drug name | Category |
| --- | --- | --- |
| N01BX04  N03AX12  N03AX16  N06AA09  N06AX21 | Capsicin  Gabapentin  Pregabalin  Amitriptylin  Duloxetin | Analgesics against neuropathic pain |
| J05B01  J05B09  J05B11 | Acyclovir  Famciclovir  Valacyclovir | Antiviral drugs |
| A07EA  L01AA01  L01AA02  L01AB01  L04A  H02AB  R03BA | Glucocorticoids, local treatment  Cytostatica  Cytostatica  Cytostatica  Selective immunosuppressants  Glucocorticoids  Glucocorticoids, inhalations | Immunosuppressive drugs |
| S01A-S01C | Anti-infective and anti-inflammatory | Ocular complication drugs |

Suppl. Table 2. ATC codes used for drugs included in the study.

**SUPPLEMENTARY TABLE 3**

VALIDATION OF HERPES ZOSTER DIAGNOSIS

The pre-defined criteria for herpes zoster were as follows: redness, grouped blisters/vesicles, pain/itching/burning sensation in the area, localized to a dermatome (defined dermatome and/or description + respecting of the midline), and previous herpes zoster episode in the same area (but not judged as recurrent herpes simplex).

If a patient was described in the chart records to have 3 or more of these symptoms (maximum of 5), the herpes zoster diagnosis was considered to be verified. Only a mention/report of “classic herpes zoster-like appearance” without any more specification was not accepted as a verified case; but these cases were listed as probable. If only 2 symptoms were recorded, and/or the original clinical report mentioned uncertainty whether herpes zoster was the only possible diagnosis, the diagnosis of herpes zoster was considered as unclear. If only one or no symptom matched, or if another clinical diagnosis such as vesicular eczema or primary staphylococci skin infection was considered, the diagnosis was considered to be unlikely. In cases of obvious misclassification, wrongful diagnosis coding or other, the diagnosis was considered as incorrect.

In almost all cases, the herpes zoster diagnosis was made upon clinical presentation, and only rarely was testing for VZV performed. In one case, 3 symptoms were described (redness, grouped blisters, intense pain) but the blisters crossed the midline (thus not conforming to clear dermatome spread) and the original clinician was unsure of the diagnosis. Since the VZV serology and sequencing results from this patient were negative, the diagnosis was thus considered as unclear.

**SUPPLEMENTARY TABLE 4**

|  |  | Men | | Women | | Both sexes | |
| --- | --- | --- | --- | --- | --- | --- | --- |
|  |  | **N** | **%** | **N** | **%** | **N** | **%** |
| 2008 | 0-49 | 449 | 28.2 | 530 | 22.9 | 979 | 25.0 |
|  | 50-54 | 81 | 5.1 | 161 | 6.9 | 242 | 6.2 |
|  | 55-59 | 131 | 8.2 | 180 | 7.8 | 311 | 7.9 |
|  | 60-64 | 168 | 10.5 | 223 | 9.6 | 391 | 10.0 |
|  | 65-69 | 178 | 11.2 | 240 | 10.3 | 418 | 10.7 |
|  | 70-74 | 183 | 11.5 | 263 | 11.3 | 446 | 11.4 |
|  | 75-79 | 185 | 11.6 | 251 | 10.8 | 436 | 11.1 |
|  | 80+ | 220 | 13.8 | 471 | 20.3 | 691 | 17.7 |
|  |  | 1,595 | 100 | 2,319 | 100 | 3,914 | 100 |
| 2009 | 0-49 | 538 | 31.3 | 628 | 24.1 | 1,166 | 27.0 |
|  | 50-54 | 89 | 5.2 | 182 | 7.0 | 271 | 6.3 |
|  | 55-59 | 109 | 6.3 | 226 | 8.7 | 335 | 7.7 |
|  | 60-64 | 190 | 11.1 | 268 | 10.3 | 458 | 10.6 |
|  | 65-69 | 209 | 12.2 | 281 | 10.8 | 490 | 11.3 |
|  | 70-74 | 179 | 10.4 | 252 | 9.7 | 431 | 10.0 |
|  | 75-79 | 158 | 9.2 | 245 | 9.4 | 403 | 9.3 |
|  | 80+ | 247 | 14.4 | 524 | 20.1 | 771 | 17.8 |
|  |  | 1,719 | 100 | 2,606 | 100 | 4,325 | 100 |
| 2010 | 0-49 | 669 | 33.2 | 821 | 27.0 | 1,490 | 29.5 |
|  | 50-54 | 107 | 5.3 | 202 | 6.6 | 309 | 6.1 |
|  | 55-59 | 151 | 7.5 | 256 | 8.4 | 407 | 8.0 |
|  | 60-64 | 202 | 10.0 | 328 | 10.8 | 530 | 10.5 |
|  | 65-69 | 244 | 12.1 | 297 | 9.8 | 541 | 10.7 |
|  | 70-74 | 209 | 10.4 | 323 | 10.6 | 532 | 10.5 |
|  | 75-79 | 160 | 7.9 | 273 | 9.0 | 433 | 8.6 |
|  | 80+ | 272 | 13.5 | 543 | 17.8 | 815 | 16.1 |
|  |  | 2,014 | 100 | 3,043 | 100 | 5,057 | 100 |

Suppl. Table 4. Age and gender distribution in cases with herpes zoster in Västra Götaland county year during 2008-2010, by calendar year.

**SUPPLEMENTARY TABLE 5**

|  |  | Men | | Women | | Both sexes | |
| --- | --- | --- | --- | --- | --- | --- | --- |
|  |  | N | % | N | % | N | % |
| 2008-2010 | 0-49 | 1,528,685 | 65.1 | 1,458,653 | 61.8 | 2,987,338 | 63.5 |
|  | 50-54 | 148,901 | 6.3 | 145,998 | 6.2 | 294,899 | 6.3 |
|  | 55-59 | 144,172 | 6.1 | 143,923 | 6.1 | 288,095 | 6.1 |
|  | 60-64 | 152,793 | 6.5 | 151,346 | 6.4 | 304,139 | 6.5 |
|  | 65-69 | 123,509 | 5.3 | 124,284 | 5.3 | 247,793 | 5.3 |
|  | 70-74 | 88,386 | 3.8 | 96,662 | 4.1 | 185,048 | 3.9 |
|  | 75-79 | 67,920 | 2.9 | 84,240 | 3.6 | 152,160 | 3.2 |
|  | 80+ | 92,955 | 4.0 | 155,458 | 6.6 | 248,413 | 5.3 |
|  |  | 2,347,321 | 100 | 2,360,564 | 100 | 4,707,885 | 100 |
| 2008 | 0-49 | 506,728 | 65.3 | 483,261 | 61.8 | 989,989 | 63.5 |
|  | 50-54 | 49,357 | 6.4 | 48,508 | 6.2 | 97,865 | 6.3 |
|  | 55-59 | 48,816 | 6.3 | 48,523 | 6.2 | 97,339 | 6.2 |
|  | 60-64 | 51,140 | 6.6 | 50,617 | 6.5 | 101,757 | 6.5 |
|  | 65-69 | 38,576 | 5.0 | 39,150 | 5.0 | 77,726 | 5.0 |
|  | 70-74 | 28,402 | 3.7 | 31,445 | 4.0 | 59,847 | 3.8 |
|  | 75-79 | 22,664 | 2.9 | 28,310 | 3.6 | 50,974 | 3.3 |
|  | 80+ | 30,794 | 4.0 | 51,839 | 6.6 | 82,633 | 5.3 |
|  |  | 776,477 | 100 | 781,653 | 100 | 1,558,130 | 100 |
| 2009 | 0-49 | 509,371 | 65.1 | 486,253 | 61.8 | 995,624 | 63.4 |
|  | 50-54 | 49,799 | 6.4 | 48,778 | 6.2 | 98,577 | 6.3 |
|  | 55-59 | 47,833 | 6.1 | 47,793 | 6.1 | 95,626 | 6.1 |
|  | 60-64 | 51,274 | 6.6 | 50,673 | 6.4 | 101,947 | 6.5 |
|  | 65-69 | 41,038 | 5.2 | 41,254 | 5.2 | 82,292 | 5.2 |
|  | 70-74 | 29,597 | 3.8 | 32,246 | 4.1 | 61,843 | 3.9 |
|  | 75-79 | 22,617 | 2.9 | 28,157 | 3.6 | 50,774 | 3.2 |
|  | 80+ | 30,953 | 4.0 | 51,822 | 6.6 | 82,775 | 5.3 |
|  |  | 782,482 | 100 | 786,976 | 100 | 1,569,458 | 100 |
| 2010 | 0-49 | 512,586 | 65.0 | 489,139 | 61.8 | 1,001,725 | 63.4 |
|  | 50-54 | 49,745 | 6.3 | 48,712 | 6.2 | 98,457 | 6.2 |
|  | 55-59 | 47,523 | 6.0 | 47,607 | 6.0 | 95,130 | 6.0 |
|  | 60-64 | 50,379 | 6.4 | 50,056 | 6.3 | 100,435 | 6.4 |
|  | 65-69 | 43,895 | 5.6 | 43,880 | 5.5 | 87,775 | 5.6 |
|  | 70-74 | 30,387 | 3.9 | 32,971 | 4.2 | 63,358 | 4.0 |
|  | 75-79 | 22,639 | 2.9 | 27,773 | 3.5 | 50,412 | 3.2 |
|  | 80+ | 31,208 | 4.0 | 51,797 | 6.5 | 83,005 | 5.3 |
|  |  | 788,362 | 100 | 791,935 | 100 | 1,580,297 | 100 |

Suppl. Table 5. Age and gender distribution of the general population in Västra Götaland county year during 2008-2010, in total and by calendar year.

* Age-standardized to the Swedish population 2010.
